# Antioxidant Capacity and HPLC-DAD-MS Profiling of Chilean Peumo (*Cryptocarya alba*) Fruits and Comparison with German Peumo (*Crataegus monogyna*) from Southern Chile

**DOI:** 10.3390/molecules18022061

**Published:** 2013-02-05

**Authors:** Mario J. Simirgiotis

**Affiliations:** Laboratorio de Productos Naturales, Departamento de Química, Universidad de Antofagasta, Antofagasta, Casilla 170, Antofagasta, 1240000, Chile; E-Mail: mario.simirgiotis@uantof.cl; Tel.: +56-55-637-229; Fax: +56-55-637-457

**Keywords:** *Cryptocarya alba*, *Crataegus monogyna*, *peumo*, HPLC-MS, *C*-glycosyl flavonoids, phenolic compounds, flavones, anthocyanins, phenolic acids, Chilean edible fruits, antioxidants

## Abstract

Liquid chromatography (LC) coupled with UV detection and electrospray ionization (ESI) tandem mass spectrometry (MS/MS) was used for the generation of chemical fingerprints and the identification of phenolic compounds in peumo fruits and aerial parts from southern Chile. Thirty three compounds (19 of these detected in *C. alba* and 23 in *C. monogyna*) were identified, mainly flavonoid glycosides, phenolic acids, anthocyanins and flavonoid aglycons. Total phenolic content and total flavonoid content was measured for both species, and were higher in the extracts from *C. monogyna* fruits and aerial parts than extracts from *C. alba*. The fruits of *Cryptocarya alba* (Chilean peumo) presented high antioxidant capacity (9.12 ± 0.01 μg/mL in the DPPH assay), but was three times lower to that of *Crataegus monogyna* (German peumo) (3.61 ± 0.01 μg/mL in the DPPH assay).

## 1. Introduction

*Cryptocarya alba* (Chilean peumo), is a shade-tolerant evergreen tree endemic of Chile, distributed from Coquimbo province (IV Region) to Valdivia province (XIV Region) mainly inhabiting streams and humid shady valleys in the forest. It produces edible red-colored berries, called *peumos*, collected wild and consumed by the Mapuche Amerindians since pre-Colombian times. It is considered a threatened species in some areas of Chile, mainly due to overexploitation and habitat destruction [1]. The essential oil of this species was reported to be composed mainly of *p*-cimol and 1-terpinen-4-ol [2] while the α-pyrone cryptofolione and a cryptofolione derivative were the only two compounds isolated from the edible fruits [3].

**Figure 1:**
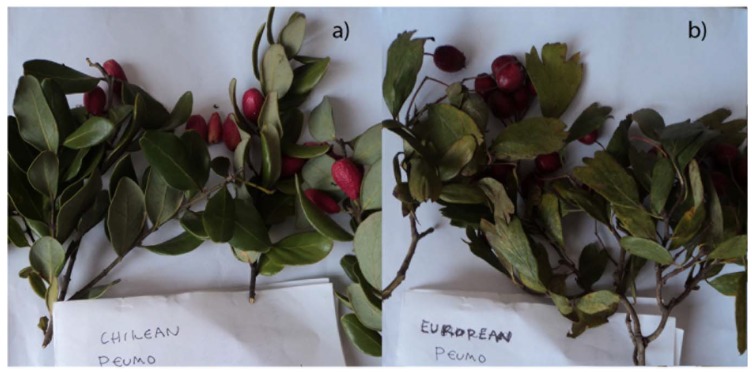
Pictures of (**a**) *Chilean peumo*; (**b**) *German peumo* collected in Re-Re, Chile.

The genus Crataegus is the largest genus among the subfamily Maloideae in the family Rosaceae which comprises 265 species, which are generally known as the hawthorns [4]. The Chilean hawthorn (*Crataegus monogyna* Jacq. (Lindt.) local name German peumo, peumo Alemán or Majuelo) is a thorny European shrub introduced to Chile and widely used as sedative, diuretic, anti-inflammatory and cardiotonic [5,6] which is prescribed by the *Pharmacopoeia Europaea* and recommended by the World Health Organization [7]. There are several reports the antioxidant capacity of and phenolic compounds present in several hawthorn species, including *C. monogyna*, which were analyzed by HPLC-MS [5,8]. However, the fruits from both species called peumo in Chile and are similar in appearance (Figure 1), yet the species are not related, even though the fruits look similar and are used for edible purposes in Chile, thus a chemical comparison and HPLC fingerprint of phenolics from both species collected in the same location (Southern hemisphere) could be a valuable tool for the differentiation of the different species and prove the health benefits of the fruits. In the present study we assessed the qualitative and quantitative phenolic profile of both edible fruits (*C. alba* and *C. monogyna*) called *peumo* in Chile by spectroscopic and spectrometric methods, evaluated their antioxidant power and compared the phenolic content with the leaves of both species. The phenolic compounds of aerial parts and fruits of the *peumos* were investigated by high-performance liquid chromatography paired with UV photodiode array and electrospray ionization ion trap tandem mass spectrometry detectors (HPLC-DAD-ESI/MS-MS).

## 2. Results and Discussion 

### 2.1. Total Phenolic, Total Flavonoid Content and Antioxidant Power of Peumo Fruits and Aerial Parts

Dietary antioxidants have been shown to be effective scavengers of harmful free radicals, preventing the oxidation of biomolecules, such as DNA and low-density lipoprotein [9,10]. Fruits and vegetables are a good source of dietary antioxidants, such as vitamin E, vitamin C and β-carotene. The best-known phytochemical antioxidants are traditional nutrients; However, the contribution of some of these nutrients and/or vitamins in different edible fruits analyzed was estimated as being lower than 15 percent [11]. The antioxidant properties of fruits and vegetables are maily due to the polyphenolic content, and several cross-cultural epidemiological studies have supported the chemoprotective properties of polyphenolics [12,13,14]. In this work methanolic extracts of fruits and leaves from Chilean peumo (*Cryptocarya alba*) and German peumo (*Crataegus monogyna*) collected in Re-Re, Chile were evaluated for antioxidant power by the DPPH scavenging activity and the ferric reducing antioxidant power assay (FRAP) and the results were compared. Both fruits showed high antioxidant power but the leaves presented the highest activity (Table 1). The fruits of *C. alba* showed total phenolic content of 17.70 ± 0.02 mg GAE (gallic acid equivalents) per g dry material. This value is 1.6 times lower than the content in *C. monogyna* fruits (28.30 ± 0.02 mg GAE/g dry material), collected in the same location. The aerial parts showed similar trend but for *C. alba* the value was 5.65 times higher (100.12 ± 0.83 mg GAE/g dry material), than its fruits, while for *C. monogyna* was 4 times higher (114.38 ± 1.62 mg GAE/g dry material), than its fruits. German peumo fruits (*C. monogyna*) also showed a higher value in total flavonoids (8.77 ± 0.00 mg QE (quercetin equivalents)/g dry material) than Chilean peumo (*C. alba*) fruits (8.22 ± 0.04 mg QE/g dry material), while the highest content of flavonoids was found in *C. monogyna* aerial parts (64.9 ± 0.00 mg QE/g dry material). *C. monogyna* fruits and aerial parts showed higher DPPH scavenging capacity (3.61 ± 0.01 and 3.34 ± 0.38 µg/mL, respectively, Table 1) and higher ferric reducing antioxidant power (85.65 ± 0.09 and 95.05 ± 0.15 µmol TE(trolox equivalents)/g, respectively, Table 1) than *C. alba* fruits and aerial parts. The antioxidant activities of polyphenolic compounds are mainly due to their ability to act as hydrogen donors, reducing agents, singlet oxygen quenchers and radical scavengers [9,10].

As reported here, the antioxidant activity of fruits and aerial parts significantly increases with high total polyphenol and flavonoid contents, however no association could be found between both antioxidant assays for these species (FRAP and DPPH, R^2^ = 0.283) and between TPC and DPPH reduction was observed positive correlation (R^2^ = 0.420), but it was not significant, as well as between FRAP and TFC (R^2^ = 0.364) and between TPC and TFC (R^2^ = 0.570) at *p* < 0.05. The low linear relationship or low correlation between the antioxidant assays and phenolic or flavonoid content as published for other plants [15,16,17,18] can be due to the different antioxidant capacity (The FRAP assay is based on the ability of the substance to reduce Fe^3+^ to Fe^2+^ while the DPPH assay the hydrogen donating capacity to scavenge DPPH radicals) or different redox properties of the mixtures of antioxidant compounds found in the organic extracts. The fruits of *C. monogyna* from Chile showed better DPPH scavenging activity than that reported for a sample from Portugal (15 ± 1% scavenging activity at 100 μg/mL) [19], but the content of phenolics and flavonoids were lower than that reported (83 ± 2 and 51 ±14 mg GAE) for that fruit sample [19].

### 2.2. HPLC DAD and MS Analysis of Phenolic Compounds from Edible Peumo Fruits and Aerial Parts

In the last years, several biological samples such as plant and fruit extracts containing mixtures of phenolic compounds have been analyzed with the use of hyphenated techniques such as liquid chromatography (HPLC, UPLC) coupled to DAD or PDA, (photodiode array detectors), and time of flight (ToF) or electrospray ionization-ion trap (ESI) mass spectrometers [20,21]. In this context we have analyzed using these precise tools several South American fruits including the white strawberry (*Fragaria chiloensis*) [22] the mountain papaya (*Vasconcellea pubescens*) [23], as well as several Mapuche Amerindian’s herbal medicines [19,24]. 

**Figure 2:**
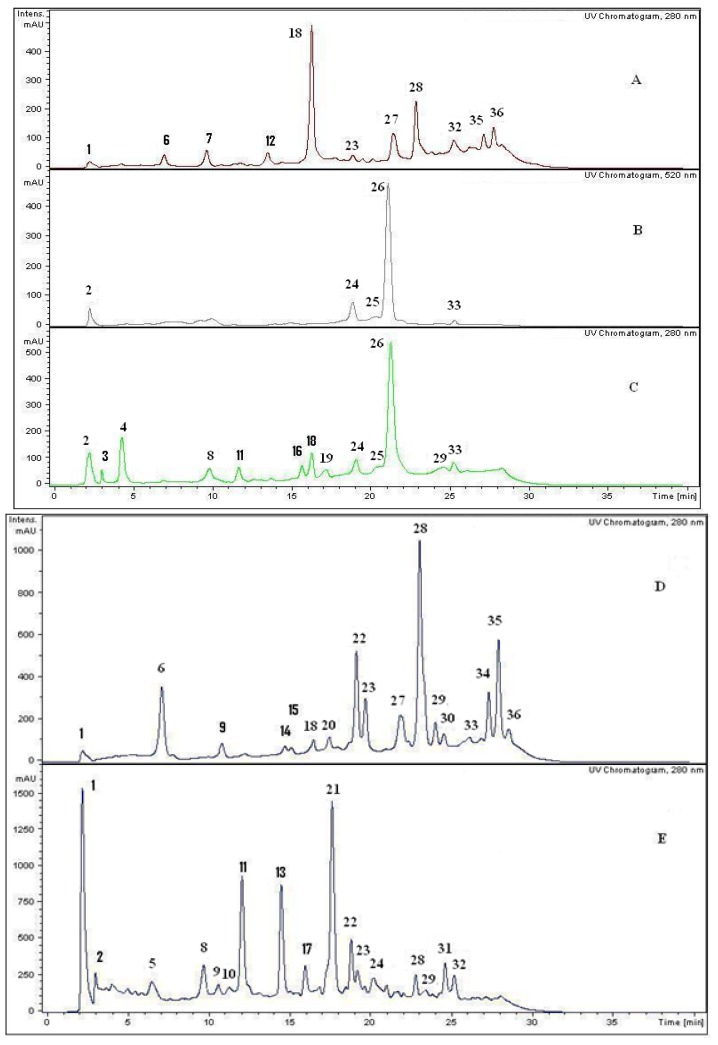
HPLC–DAD chromatograms at 280 nm of the MeOH extracts of: **A**: *C. alba* fruits; **B**: *C. monogyna* fruits (at 520 nm). **C**: *C. monogyna* fruits, **D**: *C. alba* aerial parts; **E**: *C. monogyna* aerial parts, Peak numbers refer to Table 1.

**Figure 3:**
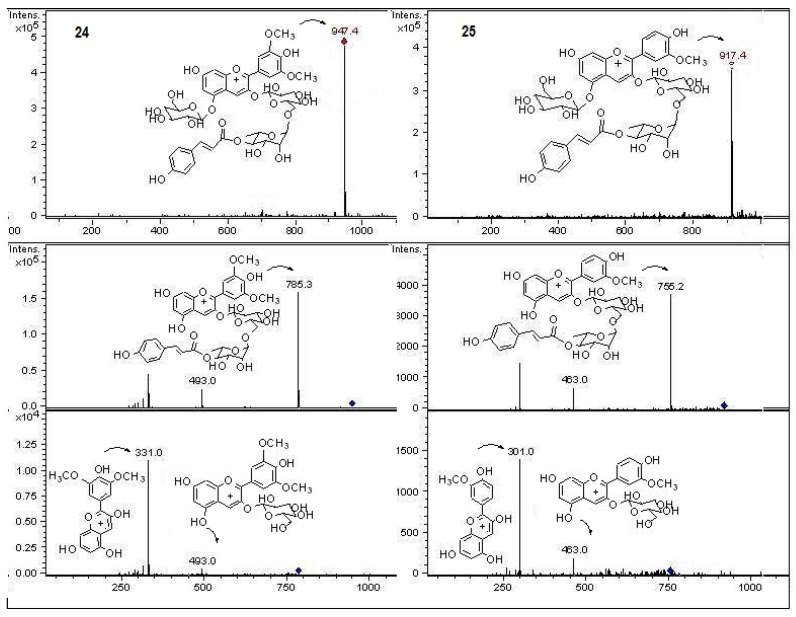
Structures, fragmentation, full ESI-MS and MS-MS spectra of peaks **24**, and **25**.

**Figure 4:**
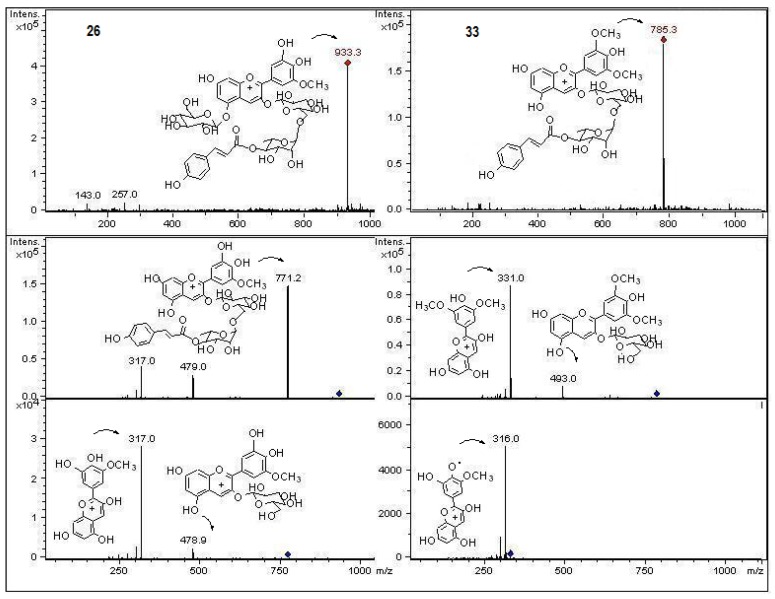
Structures, fragmentation, Full ESI-MS and MS-MS spectra of peaks **26** and **33**.

**Figure 5:**
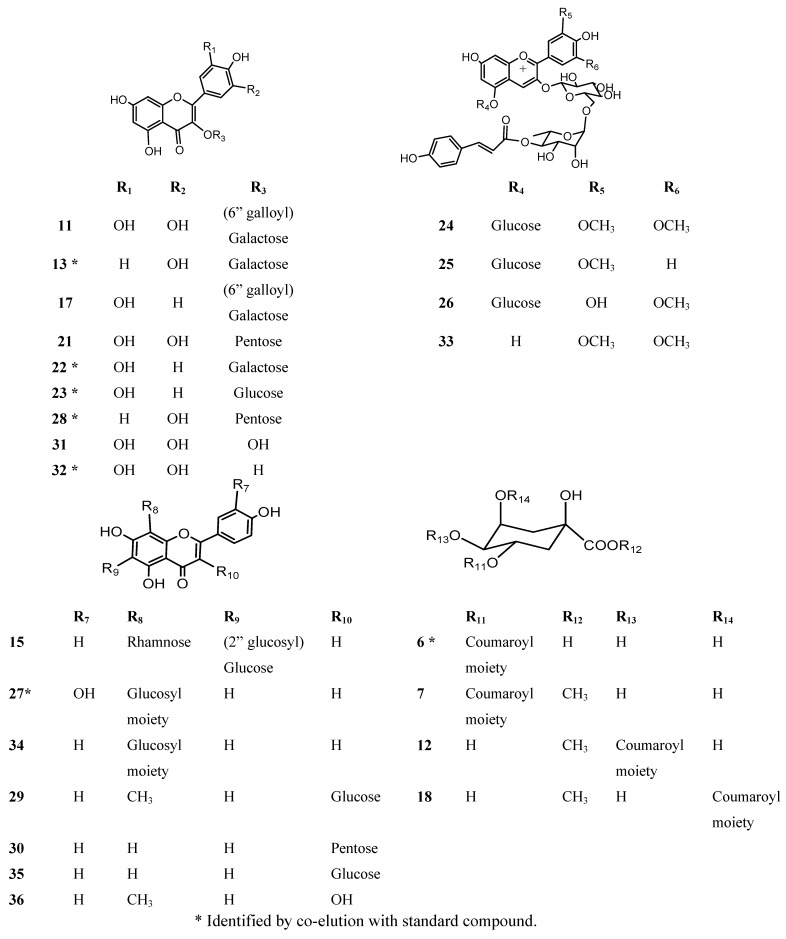
Proposed structures of flavonoids, anthocyanins and phenolic acids derivatives from *peumo* fruits identified by HPLC-DAD-ESI-MS.

**Figure 6:**
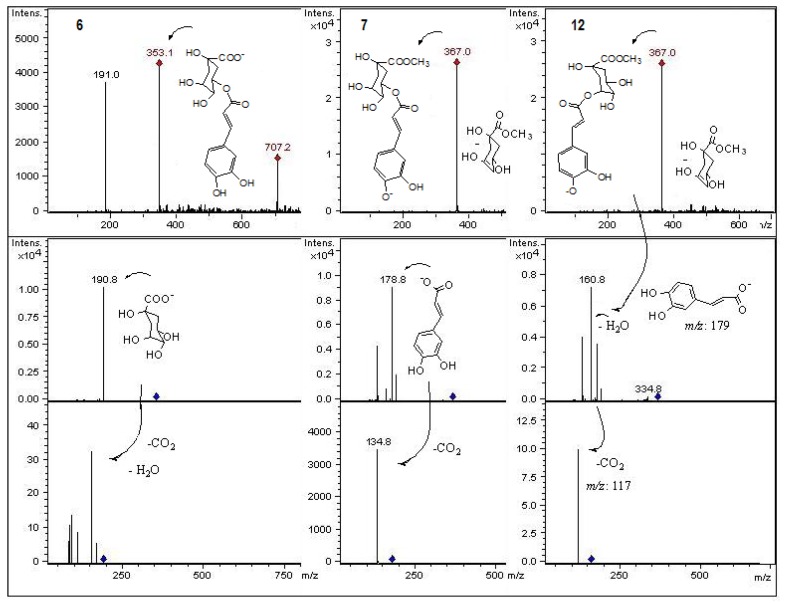
Structures, fragmentation, full ESI-MS and MS-MS spectra of peaks **6**, **7** and **12**.

**Figure 7:**
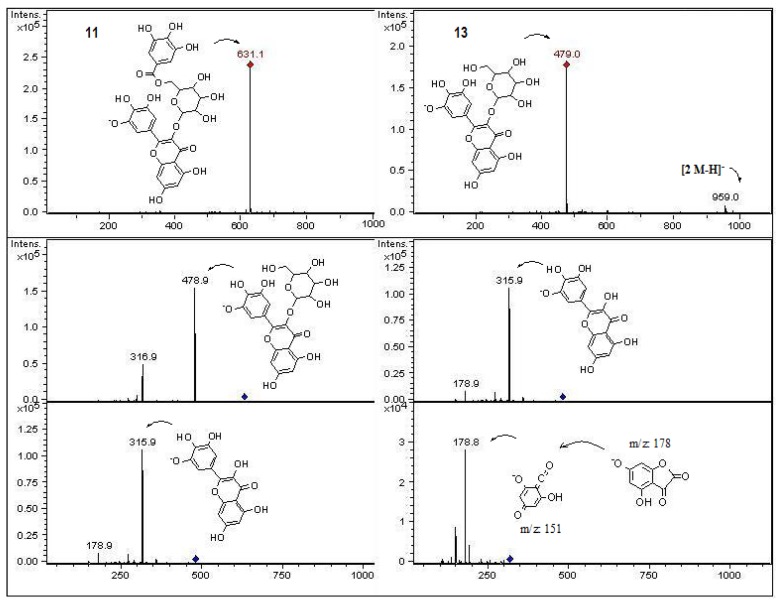
Structures, fragmentation, full ESI-MS and MS-MS spectra of peaks **11** and **13**.

**Figure 8:**
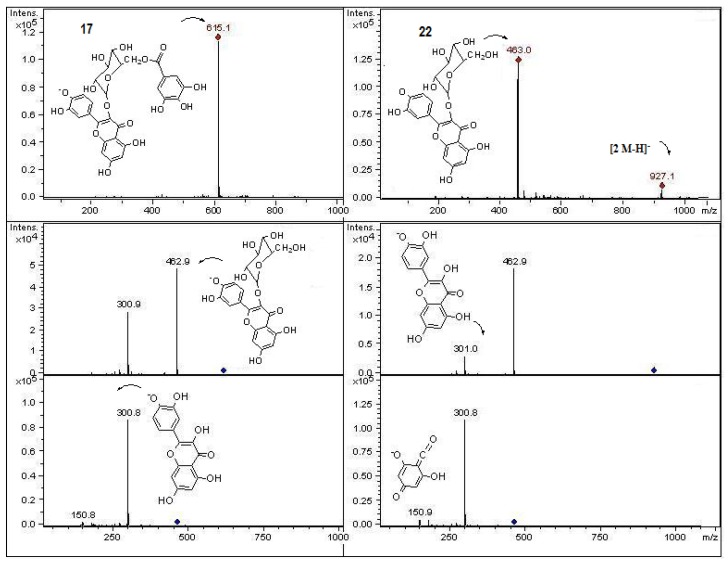
Structures, fragmentation, full ESI-MS and MS-MS spectra of peaks **17** and **22**.

**Figure 9:**
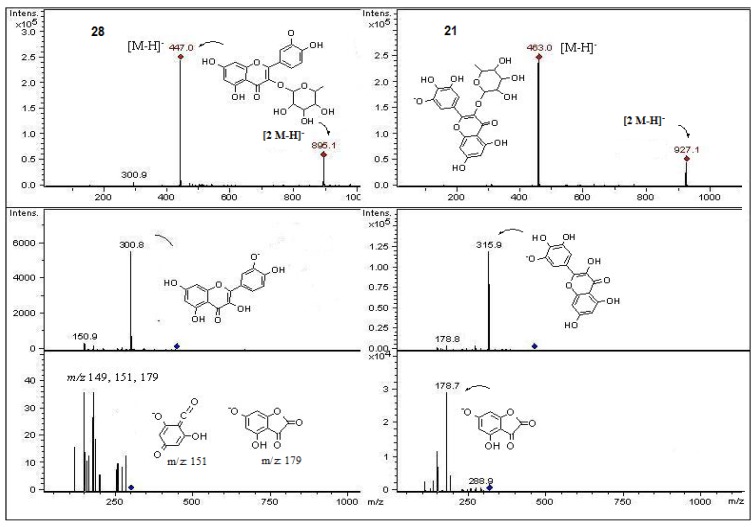
Structures, fragmentation, full ESI-MS and MS-MS spectra of peaks **21** and **28**.

**Figure 10:**
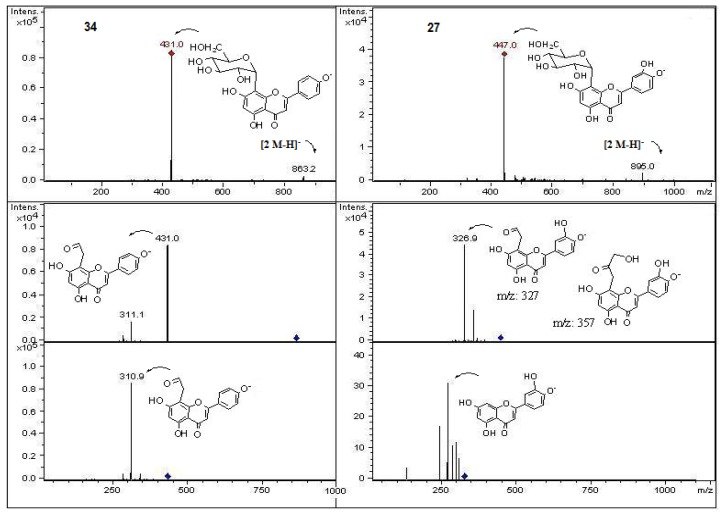
Structures, fragmentation, full ESI-MS and MS-MS spectra of peaks **27** and **34**.

**Figure 11:**
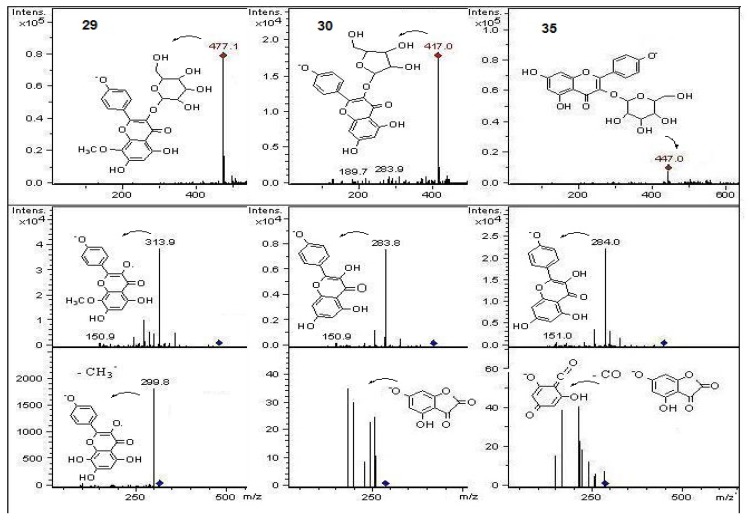
Structures, fragmentation, full ESI-MS and MS-MS spectra of peaks **29**, **30** and **35**.

In the present work and following our chemical studies on South American fruits [22,23] phenolic compounds that might be responsible [22] for the antioxidant capacity of the extracts from both peumo plants (*C. monogyna* and *C. alba*) with edible fruits growing in the VIII region of Chile were identified by HPLC using UV/visible (DAD) and tandem mass spectrometry detectors (ESI-MS-MS). For this purposes the methanolic extracts (see experimental) were injected into the HPLC system to obtain the HPLC-DAD chromatograms (Figure 2). For mass spectrometry analysis all compounds were detected in both ESI positive and negative modes. Since both fruits have a red-brown color and taking into account that the orange or red pigmentation of fruits were due generally to anthocyanins (as in blueberries, strawberries, cherries, *etc.*) or carotenoids (as in tomato, carrots, chiles, physalis, *etc.*) we searched for these compounds in the fruits under study. We found several anthocyanin derivatives (Figure 3 and Figure 4) that can be responsible for the red pigmentation in *Crataegus monogyna* (German peumo) fruits. However, we were not able to find any of those pigments (anthocyanins or carotenoids) in detectable amounts in *Cryptocarya alba* (Chilean peumo) fruits. The color of the peel of this species can thus be produced by tannins or a combination of other compounds detected in this species, since we found several groups of flavanols, *C*- and *O*-glycoside flavonoids and phenolic acids (Table 2). The mobile phase used was acidic in order to avoid the broadening of peaks due to the presence of the deprotonated form of the acid groups (carboxylic and phenolic) and to improve the retention of those compounds in the HPLC column. In addition, anthocyanins are stable in the flavilium form at a pH 1–4, so these compounds were detected in ESI positive mode, while the other phenolic compounds were detected in negative mode. In particular using the ESI ion trap detector, we could analyze cross-ring cleavages of sugar residues of three *C*-glycosyl flavones which produced main MS ions [25] that allowed differentiation with several *O*-glycosyl flavones detected (Table 2). The HPLC DAD fingerprints from the methanolic extracts of the fruits and leaves of both species are shown in Figure 2, the structures of the tentatively identified compounds are presented in Figure 5 and MS spectra are shown in Figure 5, Figure 6, Figure 7, Figure 8, Figure 9, Figure 10 and Figure 11. In this study we identified or tentatively identified 4 anthocyanins (peaks **24**–**26** and **33**), five flavanols and some flavanol derivatives (peaks **1**, **5**, **8**, **9** and **20**), two flavonol aglycones (peaks **32** and **36**), three flavonol *C*-glycosides (peaks **15**, **27** and **34**), eight phenolic acids and some of their derivatives (peaks **2**–**4**, **6**, **7**, **10**, **12** and **18**), twelve flavonoid *O*-glycosides (peaks **11**–**13**, **16**, **17**, **21**–**23**, **28**–**30** and **35**) and among those, peaks **11** and **17** were identified as galloyl derivatives. The HPLC-DAD and ESI identification of all phenolic compounds in peumo fruits and aerial parts is explained above. 

#### 2.2.1. Phenolic Acids and Related Phenolic Compounds

Peak **2** with a molecular anion at *m/z* 191 was identified as quinic acid (MS^2^ at *m/z* 110), while peak **6** was assigned as chlorogenic acid (5-*O*-caffeoyl quinic acid, Figure 6) [26] by co-elution with authentic compound. Peak **7** present in the same fruits, with a MW of 368 a.m.u. could be assigned as feruloyl quinic acid [27], however the presence of an entire caffeic acid ion at *m/z* 179 (with MS^3^ at *m/z* 135) instead of a quinic acid ion at *m/z* 191 in MS experiments led to the assignment of the compound as methyl (5-caffeoyl)quinate (Figure 6). Peaks **12** and **18** with the same UV and MS characteristics as peak 7 could be assigned as the other isomers of this compound, methyl (3-caffeoyl)quinate (Figure 6) and methyl (4-caffeoyl)quinate, respectively [28]. Peak **10** was assigned as the hydroxycinnamic acid derivative sinapoyl glucose [29].

#### 2.2.2. Flavan-3-ols (Catechins and Proanthocyanidins)

Peaks **5** and **9** were identified as catechin and its isomer epicatechin by comparison of retention time, UV and MS data with authentic compounds (diagnostic ESI-MS fragments at *m/z* 245, 205 and 179) [30]. Peaks **8** and **20** were identified as epigallocatechin gallate [31] and procyanidin B1 [32], respectively, by spiking experiments using authentic compounds. 

#### 2.2.3. Flavonoids

In mass spectrometry, *C*-glycosyl flavones experiment cross-ring cleavages of sugar residues yielding main signals (ions produced by losses of 60, 90 and 120 a.m.u) [25,33] that allowed differentiation with *O*-glycosyl flavones (losses of 162 a.m.u. for hexose, 146 a.m.u. for rhamnose and 132 a.m.u. for pentose moieties, respectively) [27]. In this work we report *C*- (peaks **15**, **27** and **34**) and *O*- glycosyl flavones (Peaks **11**, **13**, **15**–**17**, **21**–**23**, **28**–**30** and **35**). For the *C*-glycosides (Figure 9 and Figure 10) ESI-MS data was in agreement with the proposed fragmentation [34]. Peak **11** (Figure 7) with UV data 257, 292 and 361 nm, pseudomolecular ion at *m*/*z* 631 and MS-MS ions at 479 and 317 a.m.u. was identified as myricetin-3-*O*- (6” galloyl) galactoside [35] and peak **13** (Figure 7) identified as myricetin-3-*O*-galactose (myricitrin) by comparison of retention time and spectral characteristics with standard compound. Peaks **15** (anion at *m/z* 739) could be assigned as the kaempferol triglycoside robinin (kaempferol 3-*O*-robinobioside-7-*O*-rhamnoside) however no characteristic robinin daughter signal at *m/z* 593 (kaempferol 3-*O*-robinobioside) was found in the MS-MS spectra [36]. Instead, a MS^2^ fragment at 577 a.m.u. was indicative of a loss of a hexose moiety (739-162), which produced characteristic di-*C*-glycoside fragments at *m/z* 459 and 339 leading the assignment of the compound as an apigenin (2” hexoside) 6-*C*- hexosyl, 8-*C*-rhamnoside.

Peak **16** with UV data corresponding to a quercetin derivative and a [M−H]^−^ ion at *m/z* 637 which produced MS ions at *m/z* 491 (loss of glucose) and 329 (loss of rutinose) which was in concordance for the MS data reported for the flavone quercetin 7,4′-dimethyl ether or isorhamnetin 7 methyl ether [37] and thus this compound was tentatively identified as the derivative quercetin 7,4′-dimethyl ether- 3-*O*- rutinose. Peak **17** showed and [M−H]^−^ ion at *m/z* 615, and a MS^2^ ion at *m*/*z* 463 (isoquercitrin) (Figure 8) [38] produced by loss of a gallic acid unit [39] which fragmented to an MS^3^ ion at *m*/*z* 301 (deprotonated quercetin, MS^4^ ions at *m/z* 179, 151). UV spectral data of this compound is consistent with the proposed flavonoid structure quercetin-3-*O*-(6′′ galloyl) glucoside [26,40]. Peaks **21**–**24** showed a molecular anion at *m*/*z* 463. However, peak **22** was identified as hyperoside (quercetin 3-*O*-galactose, Figure 8) and peak **23** as isoquercitrin (quercetin 3-*O*- glucose), which were identified previously in hawthorn [5,6], by comparison with authentic compounds, while peak **21** (UV max. 257 and 360 nm) was identified as myricetin-3-*O*-rhamnose (Figure 9). 

Peaks **27**, **28** and **35** all with a [M−H]^−^ ion at *m/z* 447 were assigned according to UV and mass spectral data (Table 2) as luteolin 8-C-*β*-D-glucopiranoside (orientin), quercetin pentoside (Figure 9 and Figure 10) and kaempferol-glucoside [6] (Figure 11), respectively. The C-glycosyl-flavonoids identified with peaks **27** (orientin) and **34** (apigenin 8-C-*β*- D-glucopiranoside, vitexin, ([M−H]^−^ ion at *m/z* 431) [6] were identified by comparison of retention time and UV-MS spectral data (Table 1, Figure 9) with a standard compound.

Peak **29** was identified as 8-methoxykaempferol-3-*O*-glucose ([M−H]^−^ ion at *m/z* 477, with main MS^n^ fragments at *m/z* 315, 300 and 285, Figure 11) and peak **36** as the daughter compound 8-methoxy- kaempferol ([M−H]^−^ ion at *m/z* 315, with main MS^n^ fragments at 300 and 285). These kaempferol derivatives were previously reported to occur in *C. monogyna* [6]. Peak **30** ([M−H]^−^ ion at *m/z* 417 and MS^2^ at *m/z* 285 ([M−H- pentose moiety]^−^) was tentatively identified as a kaempferol pentoside (Figure 11), while peak **35** ([M−H]^−^ ion at *m/z* 447 and MS^2^ at *m/z* 285 ([M−H−hexose moiety]^−^) as a kaempferol glucoside [6] (Figure 11). Peaks **31** and **32** were identified as myricetin methyl ether ([M−H]^−^ ion at *m/z* 331 and MS^n^ ions at *m/z* 315 ([M−2H−CH_3_]^−^), 300 ([M−2H−2CH_3_]^−^) and myricetin ([M−H]^−^ ion at *m/z* 317 and MS^n^ ions at *m/z* 300 [M−H_2_O]^−^, 179 and 151), respectively. The latter compound was identified by spiking experiment with an authentic standard.

#### 2.2.4. Anthocyanins

Peaks **24**, **25** and **26** with molecular cations at *m*/*z* 947, 917 and 933, respectively, were identified as the anthocyanins malvidin, peonidin and petunidin 3-*O*-(4′′′-coumaroyl)rutinoside 5-*O*-glucoside (Figure 3 and Figure 4), triglycosylcoumaroyl phenolic compounds previously reported to occur in pigmented potatoes [41], while peak **33** with a [M+H]^+^ ion at *m/z* 785 was tentatively identified as the related anthocyanin malvidin 3-*O*-(4′′′-coumaroyl)rutinose (Figure 4). 

#### 2.2.5. Unidentified Compounds

Peak **3** was assigned as an unknown quinic acid derivative with a molecular ion at *m/z* 381, producing a quinic acid MS^2^ fragment at *m/z* 191 (MS^3^ at 110 a.m.u.). Peaks **14** and **19** with ions at 761 and 733 U and UV-vis spectra characteristic of 3 *O*-flavonols (254, 360 nm) remain unknown.

## 3. Experimental

### 3.1. General 

HPLC grade water, methanol and acetonitrile, formic acid, HCl, KCl, Folin–Ciocalteu phenol reagent, sodium acetate, aluminum chloride hexahydrate and sodium carbonate were purchased from Merck (Darmstadt, Germany). Amberlite XAD-7HP 20-60 mesh resin, quercetin, 1,1-diphenyl-2-picrylhydrazyl (DPPH^.^) and gallic acid were purchased from Sigma Chemical Co. (St. Louis, MO, USA). Isoquercitrin, myricitrin, (+)catechin, (-)epicatechin, epigallocatechin gallate, orientin, vitexin, myricetin and chlorogenic acid for HPLC analysis all with purity higher than 95% (with HPLC certificate) were purchased either from ChromaDex (Santa Ana, CA, USA) or Extrasynthèse (Genay, France).

LC-DAD analyses were carried out using a Merck-Hitachi equipment with a quaternary L-7100 pump, a L-7455 UV diode array detector, and a D-7000 chromato-integrator (LaChrom, Tokyo, Japan). A 250 × 4.6 mm i.d., 5 *μ*m, Purospher star-C18 column (Merck, Germany) set at 25 °C was used for the separation of all phenolics. Detection was carried out at 280, 354 and 520 nm, with peak scanning between 200 and 600 nm. Gradient elution was performed with water/1% formic acid (solvent A) and acetonitrile/1% formic acid (solvent B) at a constant flow rate of 1.0 mL/min. An increasing linear gradient (v/v) of solvent B was used [*t* (min), % A]: 0, 90; 4, 90; 25, 75; 40, 90. For LC-ESI-MS analysis an Esquire 4000 Ion Trap mass spectrometer (Bruker Daltoniks, Bremen, Germany) was connected to an Agilent 1100 HPLC (Agilent Technologies, Waldbronn, Germany) instrument via ESI interface. A Bruker Daltoniks 3.2 data analysis software was used for acquisition and processing. Full scan mass spectra were measured between *m/z* 150 and 2000 U in negative ion (preferred) mode. Nitrogen was used as nebulizer gas at 27.5 psi, 350 °C and at a flow rate of 8 l/min. The mass spectrometric conditions were: electrospray needle, 4000 V; end plate offset, −500 V; skimmer 1, −56.0 V; skimmer 2, −6.0 V; capillary exit offset, −84.6 V. Collision induced dissociation (CID) spectra were obtained with a fragmentation amplitude of 1.00 V (MS/MS) using ultrahigh pure helium as the collision gas. The spectroscopic measurements were performed using a Unico 2800 UV-vis spectrophotometer (Unico instruments, Co, Ltd., Shangai, China). 

### 3.2. Plant Material

The study was carried out with ripe fruits and aerial parts (leaves and stems) of *Cryptocarya alba* (Molina) Looser (local name: peumo chileno), and *Crataegus monogyna* (Molina) A. Gray (local name: peumo Alemán), which were collected by Luis Bermedo Guzmán and Mario J. Simirgiotis in Re-Re, Región del Bio-Bio, Chile in May 2011. Examples were deposited at the Laboratorio de Productos Naturales, Universidad de Antofagasta, Antofagasta, Chile, with the numbers Ca-111505-1 and Cm-111505-1, respectively. 

### 3.3. Sample Preparation

Fresh *peumo* fruits and aerial parts (leaves and stems) were separately homogenized in a blender and freeze-dried (Labconco Freezone 4.5 L, Kansas, MO, USA). One gram of lyophilized material was finally pulverized in a mortar and extracted thrice with 25 mL of 0.1 % HCl in MeOH in the dark for one hour each time. The extracts were combined, filtered and evaporated *in vacuo* (40 °C). The extracts were suspended in 10 mL ultrapure water and loaded onto a reverse phase solid phase extraction cartridge (SPE, Varian Bond Elut C-18, 500 mg/6 mL). The cartridge was rinsed with water (10 mL) and phenolic compounds were eluted with 10 mL MeOH acidified with 0.1 % HCl. The solutions were evaporated to dryness under reduced pressure to give 184.6 mg of *C. alba* fruits, 127.7 mg of *C. alba* aerial parts, 146.8 mg of *C. monogyna* fruits and 118.3 mg of *C. monogyna* aerial parts, respectively (for extraction yields see Table 1). The extracts were then dissolved in MeOH:water 7:3 (approximately 2 mg/mL) filtered through a 0.45 μm micropore membrane (PTFE, Waters) before use and 20 μl were injected into the HPLC instrument for analysis.

### 3.4. Polyphenolic Content

A precisely weighed amount of each extract (approximately 2 mg/mL) as explained in Section 3.3 was used for total phenolic (TPC) and total flavonoid (TFC) content. Extracts were dissolved in a MeOH:water 7:3 v/v solution. Appropriate dilutions were prepared and absorbance was measured using a spectrophotometer (see Section 3.1). The TPCs were determined by the Folin and Ciocalteu’s reagent method [42]. Briefly, the appropriate extract dilution was oxidized with the Folin-Ciocalteu reagent (2 mL, 10 % v/v), and the reaction was neutralized with sodium carbonate. The calibration curve was performed with gallic acid (concentrations ranging from 16.0 to 500.0 μg/mL, R^2^ = 0.999). The absorbance of the resulting blue color of the complex formed was measured at 740 nm after 30 min, and the results were expressed as mg of gallic acid equivalents per g dry material. The TFCs in the samples were determined as previously reported [43]. The absorbance of the reaction mixture (2.5 mL) was measured at 430 nm and quercetin was used as a reference for the calibration curve (concentrations ranging from 16.0 to 800.0 µg/mL, R^2^ = 0.994). Results were expressed as mg quercetin equivalents per g dry weight. Data are reported as mean ± SD for at least three replications. 

### 3.5. Antioxidant Assessment

#### 3.5.1. Bleaching of the 2,2-diphenyl-1-picrylhydrazyl (DPPH) Radical Assay

Free radical scavenging capacity was evaluated according to the method described previously [27] Briefly, aliquots of samples (100 μL) were assessed by their reactivity with a methanol solution of 100 μM DPPH. The reaction mixtures (2 mL) were kept for 30 min at room temperature in the dark. The decrease in the absorbance (n = 3) was measured at 517 nm, in a Unico 2800 UV-vis spectrophotometer (Shanghai, Unico instruments, Co, Ltd). The percent DPPH scavenging ability was calculated as: DPPH scavenging ability = (A_control_ – A _sample_/A_control_) × 100. Afterwards, a curve of % DPPH scavenging capacity *versus* concentration was plotted and IC_50_ values were calculated. IC_50_ denotes the concentration of sample required to scavenge 50 % of DPPH free radicals. The lower the IC_50_ value the more powerful the antioxidant capacity. If IC_50_ ≤ 50 μg/mL the sample has high antioxidant capacity, if 50 μg/mL < IC_50_ ≤ 100 μg/mL the sample has moderate antioxidant capacity and if IC_50_ > 200 μg/mL the sample has no relevant antioxidant capacity. In this assay, the standard antioxidant compound gallic acid showed an IC_50_ value of 1.16 μg/mL (6.81 μM). 

#### 3.5.2. Ferric Reducing Antioxidant Power (FRAP) Assay

The FRAP assay was done according to [44] with some modifications. The stock solutions included 300 mM acetate buffer pH 3.6, 10 mM TPTZ (2,4,6-tripyridyl-*s*-triazine) solution in 40 mM HCl, and 20 mM FeCl_3_·6H_2_O solution. The working solution was prepared by mixing 50 mL acetate buffer, 10 mL TPTZ solution, and 15 mL FeCl_3_·6H_2_O solution and then warmed at 37 °C before using. Tumbo fruit extracts (100 μL) were allowed to react with 2 mL of the fresh FRAP solution for 30 min in the dark. Readings of the coloured product ferrous tripyridyltriazine complex were then taken at 593 nm (n = 3). The standard curve was performed with the standard antioxidant Trolox (R^2^ = 0.9995). Results are expressed in mM TE (Trolox equivalents)/ g dry mass. 

### 3.6. Statistical Analysis

The statistical analysis was carried out using the originPro 9.0 software packages (Originlab Corporation, Northampton, MA, USA). The determination was repeated at least three times for each sample solution. Analysis of variance was performed using one way ANOVA and Tukey test (*p* values < 0.05 were regarded as significant).

## 4. Conclusions

The HPLC fingerprints showed in this work can be used to authenticate and differentiate the edible fruits of the two species called *peumo* from the VIII region of Chile, which are similar in appearance and are grown in the same location and used for similar food purposes. Furthermore, based on our LC/DAD and LC/MS experiments, the distribution of different phenolics in the two species has been analyzed and a total of 33 phenolic compounds were detected and characterized, or tentatively identified for the first time for both species from Chile (19 of those detected in *C. alba* and 23 in *C. monogyna*) many of which have not been described hitherto in these plant materials, especially for *C. alba*. The extracts obtained from *C. alba* fruits (Chilean peumo) and aerial parts showed high antioxidant capacity which is three times lower to that found for *C. monogyna* fruits, but was higher for aerial parts, which might be related with the number of phenolic compounds and total phenolic content found in these extracts. The compounds identified can be also used as biomarkers especially for *C. alba* since little research has been published for this species. The phenolic profiles of the different plant parts revealed high predominance of flavonoids, which are antioxidant compounds that modulate a variety of beneficial biological events. Therefore, *C. alba* edible fruits and aerial parts may be considered a source of important phytochemicals (mainly flavonoids and phenolic acids) with bioactive properties to be explored for pharmaceutical applications.

## Figures and Tables

**Table 1:** Total phenolic content (TPC), total flavonoid content (TFC) and ferric reducing antioxidant power (FRAP), scavenging of the free radical DPPH and percent w/w extraction yield of peumo methanolic extracts on the basis of freeze-dried starting material.

Species and plant part ^a^	TPC ^b^ (mgGAE/g)	TFC ^b^ (mgQE/g)	FRAP ^b^ (µmolTE/g)	DPPH ^b^ (IC_50_, µg/mL)	w/w extraction yield (%)
*C. alba* fruits	17.70 ± 0.02	8.22± 0.04	39.65 ± 0.04	9.12 ± 0.01	15.43
*C. monogyna* fruits	28.30 ± 0.02	8.77 ± 0.00	85.65 ± 0.09	3.61 ± 0.01	12.36
*C. alba* aerial parts	100.12 ± 0.83	15.7 ± 0.01	29.22 ± 0.04	3.92 ± 0.02	13.32
*C. monogyna* aerial parts	114.38 ± 1.62	64.9 ± 0.00	95.05 ± 0.15	3.34 ± 0.38	8.90

^a^ Data expressed as means ± standard deviation. ^b^ Means in the same column differ significantly (at *p* < 0.05) in ANOVA test. GAE: Gallic acid equivalents; QE: Quercetin equivalents; TE: Trolox equivalents.

**Table 2:** Identification of phenolic compounds in peumo fruits and leaves by LC-DAD, LC–MS and MS/MS data.

Peak #	Rt (min)	λ max (nm)	+/− ions	[M−H]^−^ (*m/z*)	[2M-H]^−^ (*m/z*)	Fragment ions (*m/z*)	POLYPHENOLS IDENTITY	SPECIES/PART
1	2.3	280	−	593		425, 289	Epigallocatechin-catechin dimer	Cral, Cryl, Cryf
2	3.0	-	−	191		110	Quinic acid	Cral, Craf
3	3.0	280	−	381		191, 110	quinic acid derivative	Cral, Craf
4	3.9	265	−	169		137, 125, 97	Gallic acid*	Craf
5	6.5	278	−	280		245, 205, 179	Catechin *	Cral
6	7.1	242, 300sh, 325	−	353		191, 110	Chlorogenic acid *	Cryl, Cryf
7	9.6	240, 295sh, 332	−	367		179, 135	Methyl -(5-caffeoyl)-quinate	Cryf
8	9.5	272	−	457	915	305	Epigallocatechin gallate *	Cral, Craf
9	10.7	280	−	289		245, 205, 179	Epicatechin *	Cryl
10	11.5	234, 295sh, 325	−	385		223, 205,	1-*O*-sinapoyl-*β*-D-glucose	Cral
11	12.0	255, 293sh, 358	−	631		479, 316,179	Myricetin-3-*O*-(6′′ galloyl) galactose	Cral, Craf
12	13.6	240, 295sh, 332	−	367	735	179, 161	Methyl (3-caffeoyl)-quinate	Cryf
13	14.4	254–362	−	479	959	316, 179	Myricetin -3-*O*-galactose (myricitrin) *	Cral
14	14.7	254, 360	−	761		609, 471, 361	Unknown flavonoid glycoside	Cryl
15	15.1	264, 335	−	739		721, 577, 435, 339	(Apigenin (2′′ hexoside) 6-*C*- hexosyl, 8-*C*-rhamnoside)	Cryl
16	15.6	254, 354	−	637		491, 329, 179, 151	Quercetin 7,4′-dimethyl ether- 3-*O*- rutinose	Craf
17	15.9	254, 290sh, 360	−	615		463, 301	Quercetin-3-*O*-(6′′ galloyl) glucoside	Cral
18	16.2	240, 295sh, 332	−	367	735	179, 135	Methyl (4-caffeoyl)-quinate	Craf
19	17.0	246, 265	−	733		671, 601	Unknown	Craf
20	17.4	278	−	577		451, 425, 407, 289	Procyanidin B1 *	Cryl
21	17.6	253, 365	−	463	927	316, 178	Myricetin 3-*O*- rhamnose	Cral
22	18.8	254, 363	−	463	927	301, 179, 151	Hyperoside *	Cral, Cryf
23	20.5	254, 360	−	463	927	301, 179, 151	Isoquercitrin *	Cral, Cryf, Cryl
24	20.7	275, 292sh, 343sh, 512	+	947		785, 493, 331	Malvidin-3-*O*-(4′′′coumaroyl)-rutinose-5-*O*- glucose	Cral, Craf
25	21.0	268, 290sh, 357sh, 503	+	917		755, 463, 301	Peonidin-3-*O*- (4′′′coumaroyl)-rutinose-5-*O*- glucose	Craf
26	18.7	275, 290sh, 343sh, 512	+	933		771, 479, 317	Petunidin-3-*O*- (4′′′coumaroyl)-rutinose-5-*O*- glucose	Craf
27	21.4	266, 292sh, 352	−	447	895	327	Luteolin 8-*C*-glucose (orientin) *	Cryl, Cryf
28	22.9	254, 364	−	447	895	300,179, 151	Quercetin -3-*O*-pentoside	Cral, Cryl, Cryf
29	23.5	265, 352	−	477		315, 300, 285	8-Methoxy- Kaempferol -3-*O*- glucose	Cral, Cryl, Craf
30	24.2	265, 352	−	417		285	Kaempferol-3-*O*-pentose	Cryl
31	24.8	254, 362	−	331		315, 300, 179, 151	Myricetin 3′ methyl ether	Cral
32	25.1	254, 360	−	317		300, 179, 151	Myricetin *	Cryl, Cral
33	26.1	275, 292sh, 343sh, 512	+	785		493, 331	Malvidin-3-*O*-(4′′′coumaroyl)-rutinose	Craf, Cryl
34	27.1	268, 335	−	431		311	Apigenin 8-*C*-glucose (vitexin) *	Cryl
35	27.7	266, 350	−	447	895	285	Kaempferol 3-*O*- glucose	Cryl, Cryf
36	28.2	266, 350	−	315		300, 285	8-Methoxy- Kaempferol	Cryl, Cryf

* Identified with authentic standards. Cral, *Crataegus* leaves, Cryl, *Cryptocarya* leaves Cryf, *Cryptocarya* fruits Craf, *Crataegus* fruits.
